# Structural properties of ultrathin SrO film deposited on SrTiO_3_

**DOI:** 10.1080/14686996.2019.1599693

**Published:** 2019-05-20

**Authors:** Tornike Gagnidze, Huan Ma, Claudia Cancellieri, Gian-Luca Bona, Fabio La Mattina

**Affiliations:** a Laboratory for Transport at Nanoscale Interfaces, Empa Swiss Federal Laboratories for Science and Technology, Dübendorf, Switzerland; b Laboratory for Joining Technologies and Corrosion, Empa Swiss Federal Laboratories for Science and Technology, Dübendorf, Switzerland

**Keywords:** Pulsed laser deposition, ultrathin film, perovskite, epitaxial film, surface termination, 10 Engineering and Structural materials, 101 Self-assembly / Self-organized materials, 202 Dielectrics / Piezoelectrics / Insulators, 212 Surface and interfaces, 306 Thin film / Coatings

## Abstract

The role of epitaxial strain and chemical termination in selected interfaces of perovskite oxide heterostructures is under intensive investigation because of emerging novel electronic properties. SrTiO${\;}_{3}$ (STO) is one of the most used substrates for these compounds, and along its <001> direction allows for two nonpolar chemical terminations: TiO_2_ and SrO. In this paper, we investigate the surface morphology and crystal structure of SrO epitaxial ultrathin films: from 1 to about 25 layers grown onto TiO${\;}_{2}$-terminated STO substrates. X-ray diffraction and transmission electron microscopy analysis reveal that SrO grows along its [111] direction with a 4% out-of-plane elongation. This large strain may underlay the mechanism of the formation of self-organized pattern of stripes that we observed in the initial growth. We found that the distance between the TiO${\;}_{2}$ plane and the first deposited SrO layer is 0.27(3) nm, a value which is about 40% bigger than in the STO bulk. We demonstrate that a single SrO-deposited layer has a different morphology compared to an ideal atomically flat chemical termination.

## GRAPHICAL ABSTRACT

**Figure:**
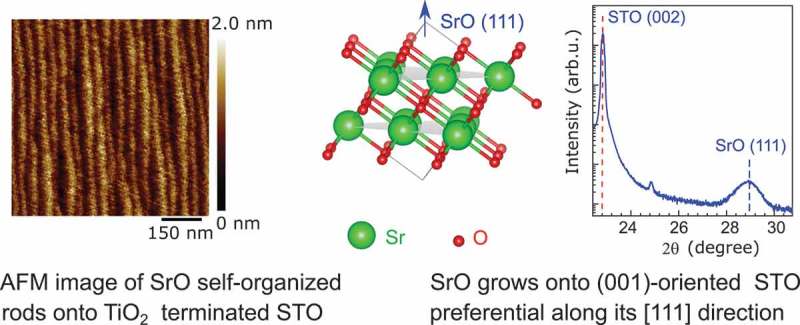

## 1. Introduction

Multilayers of thin films of transition metal perovskite oxides are under intensive investigation due to emerging novel properties observed in selected interfaces. Most of the properties of the perovskite compounds (ABO${\;}_{3}$) originate from a large oxygen octahedron containing the B cation (BO${\;}_{6}$) with a typical dimension of about 0.4 nm. Due to this octahedron, some of these compounds have a low lattice mismatch, and can be stacked in multilayer epitaxial heterostructures. At the interfaces of these structures, the connection between the oxygen octahedron [1], the polar discontinuity [2–5], and the lattice strain [6–10] can be tailored in order to enhance or even to engineer new properties with respect to the parent compounds. For instance, it has been observed that, in case of mixed termination of STO, SrRuO${\;}_{3}$ selectively grows only on the TiO${\;}_{2}$ islands, i.e. the ruthenate reproduces the TiO${\;}_{2}$ pattern present on the surface [8,9]. Another important example of a special interface phenomenon is the case of the LaAlO${\;}_{3}$/SrTiO${\;}_{3}$ (LAO/STO) epitaxial heterostructures, where two-dimensional electron gas (2DEG) forms unexpected at the interface of the two insulators [2,11,12]. This phenomenon has been observed only in case of TiO${\;}_{2}$-terminated STO substrate, and a debate about the role of polar discontinuity and strain-induced oxygen vacancies is still running [13–16].

In the rest of this manuscript, we focus on the crystal quality of the different chemical terminations of STO, which is one of the most used substrates for the growth of perovskite oxide heterostructures. Along its <001> direction, this crystal allows for two nonpolar chemical terminations: TiO${\;}_{2}$ and SrO. The first type of termination can be achieved by a well-established two-step process: first, a selective hydrogen fluoride (HF) etching of SrO followed by an oxygen annealing at high temperature (≥950°C) [17,18] which induces a reorganization of the surface in TiO${\;}_{2}$ terraces. A simple annealing in oxygen at high temperature (≤1000°C) is not capable to remove SrO and will produce a mixture of SrO and TiO${\;}_{2}$ islands [19].

Up to now a reproducible chemical treatment for the SrO terminations has not yet been achieved. It has been reported that annealing above 1300°C induces Sr extrusion from the bulk, and produces SrO islands with irregular shapes [20,21]. Only in rare cases, it has been observed that islands smaller than 20 nm have attached at the edges of the TiO${\;}_{2}$ terraces [8,22]. In this case, the distance between SrO and TiO${\;}_{2}$ planes is almost identical to the bulk value (∼0.19 nm), while in case of isolated SrO islands Takahashi et al. [23] report a value of about 0.23 nm.

Deposition of SrO monolayers by molecular beam epitaxy [23,24] or pulsed laser deposition (PLD) [2,25–27] is a good alternative to chemical treatments. In all these cases it was assumed that the deposited SrO monolayer has the same lattice parameters as bulk STO.

**Figure 1.**
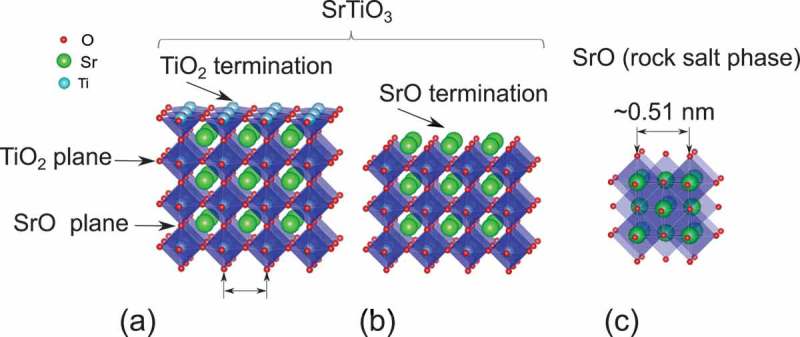
Schematic view of SrTiO${\;}_{3}$ substrate with TiO${\;}_{2}$ (a) and SrO (b) terminations. (c) Most stable phase of SrO rock salt with cubic lattice parameter of 0.51 nm [29,30].

Figure 1 shows a schematic view of SrTiO${\;}_{3}$ substrate with TiO${\;}_{2}$ (a) and SrO (b) terminations. In the ideal case, the distance between the top SrO layer and the nearest TiO${\;}_{2}$ plane is about 0.19 nm, like in the rest of the bulk of the STO. In this structure, while a hybridization of the O(2p) states with the Ti(3d) states leads to a pronounced covalent bonding for the Ti [28], the Sr${\;}^{2+}$ and O${\;}^{2-}$ ions exhibit more an ionic bonding character. Therefore, in the case of SrO termination of STO, the presence of vacuum interface could allow the Sr to organize differently respect to the bulk case, where its position is forced by the surrounding TiO${\;}_{6}$ octahedra. Moreover, the most stable phase of SrO rock salt (Figure 1(c)) has a cubic lattice parameter of 0.51 nm [29,30] which is 30% higher than STO. Therefore, during the deposition of SrO, it is expected that it will grow on a different crystal axis in order to minimize the strain with the substrate. In recent studies, it has been shown that during the deposition of SrO onto STO some defects at the interface are generated in forms of Ti atoms incorporated into SrO and missing Ti from STO [31,32]. In this manuscript, we present an investigation about the surface morphology and crystal structure of SrO epitaxial films grown onto TiO${\;}_{2}$-terminated STO substrates. Due to the large lattice mismatch with STO, we observed that SrO grows preferentially along its [111] direction, and an elongation of 4% was clearly observed by X-ray diffraction (XRD) and transmission electron microscopy (TEM) along this direction. We propose that this large strain is also responsible of self-organized patterns formed in the initial growth of SrO. Here, we demonstrate that a single SrO layer deposited onto STO shows a granular surface morphology intrinsically different compared to the atomically flat TiO${\;}_{2}$ termination.

## 2. Material and methods

**Figure 3.**
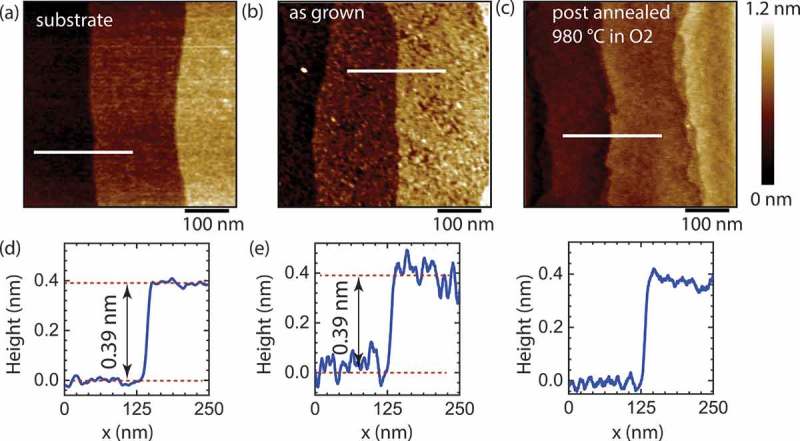
AFM surface topographies (a–c) and representative line profiles (d–f) of TiO${\;}_{2}$-terminated STO substrate (a), as-grown (b) and post-annealed (c) SrO film. The substrate shows typical step-like morphology with the height of each step of about 0.39 nm. While the pristine substrate has atomically flat terraces, the surface of the as-grown film shows a granular topology. After the annealing, the surface roughness reduces from 0.12(1) to 0.06(1) nm.

SrO films were deposited by means of PLD, using KrF excimer laser (λ = 248 nm), with laser fluency of about 1.5 J/cm${\;}^{2}$. During the PLD growth, the temperature of the substrates was fixed at 725°C with 100 mTorr oxygen pressure inside the chamber. Two different targets were prepared by pressing SrCO${\;}_{3}$ (99.995 mol%, Sigma-Aldrich, Germany) or SrO (99.95 mol%, abcr GmbH, Germany) powders and by sintering in air at 750 and 300°C, respectively. These two targets gave similar results for the film deposition; however, the SrCO${\;}_{3}$ target was more stable in a time range of about 2 months. The distance between the target and substrate was set to 45 mm for all the experiments. We used *in situ* reflection high-energy electron diffraction (RHEED) to control the evolution of the epitaxial layers during the growth of each films. Commercial (CRYSTAL GmbH, Germany) high-quality single-crystalline (001)-oriented STO was used as a substrate for films growth. The miscut angle was ≤0.1${\;}^{\circ }$ for all substrates. TiO${\;}_{2}$-terminated surfaces were obtained by typical HF etching and oxygen annealing procedure [18]. Only substrates with terraces width between 150 and 200 nm were selected for this investigation. The surface topography of the substrates and the films were studied by atomic force microscopy (AFM) (Bruker Icon3, Bruker Corporation, USA) with Tespa-V2 AFM cantilevers in tapping mode. A typical surface of TiO${\;}_{2}$-terminated STO substrate is shown on Figure 3(a). The step between each of the terraces is about 0.39 nm (Figure 3(d)), which is one unit cell of STO and indicates single chemical termination. Crystal structure of SrO film were investigated by Bruker D8 Discover XRD, and by a (TEM Jeol JEM 2200 fs, JEOL Ltd., Japan) which operates at accelerating voltage of 200 kV. Lamellas for TEM analysis were prepared by focused ion beam FEI Helios 660 (Thermo Fisher Scientific).

## 3. Results and discussion

**Figure 2.**
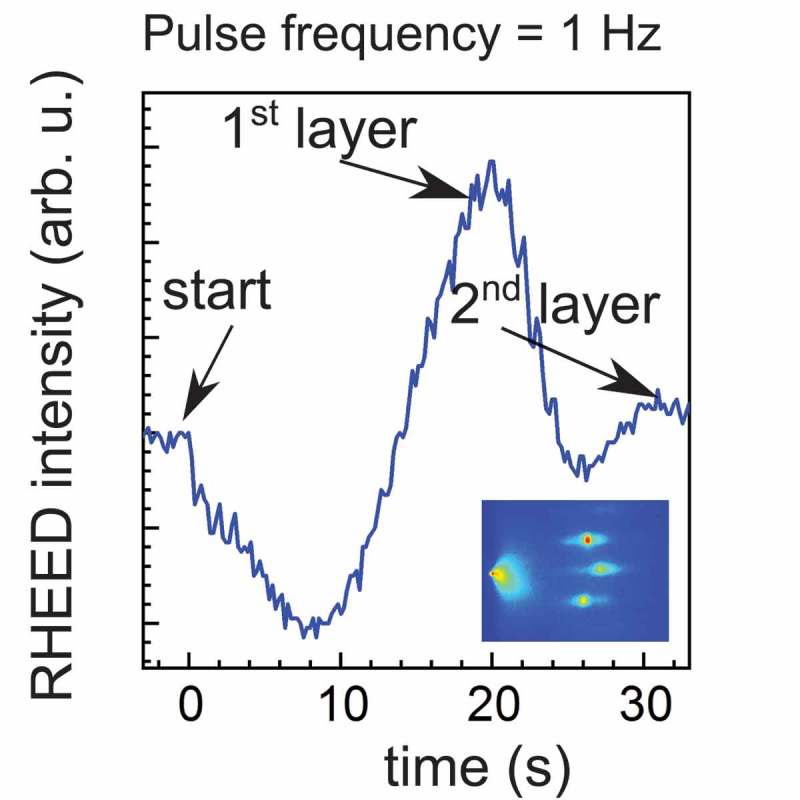
Typical RHEED intensity during the deposition of about two layers of SrO onto TiO${\;}_{2}$-terminated STO. The inset shows the initial RHEED pattern of the substrate.

Several authors report on epitaxial growth of SrO thin films in the temperature range between 600 and 800°C [2,23–27]. Some of them [23,24] show RHEED intensity oscillation during the deposition of several layers which is typical of the two-dimensional (2D) growth so called layer-by-layer mode. However, the RHEED intensity signal reported during the deposition of the first layer does not show a clear reproducible behavior in literature [2,23–27]. In Figure 2, we report a representative RHEED intensity measured over the deposition of about two layers of SrO onto TiO${\;}_{2}$-terminated STO. Here, the growth rate of SrO was estimated to be about 18 pulses per layer. This value was confirmed by TEM in the case of thicker film, shown later in this paper, and was used to scale the surface coverage for all the films presented here. In order to understand the initial growth of this layer, we should consider a low atomic surface diffusion, and the fact that the most stable phase of SrO rock salt has a cubic lattice parameter of 0.51 nm [29,30], which is about 30% higher than the STO (0.39 nm). This big lattice mismatch could produce high strain also during the growth of one individual plane of SrO. Moreover, local defects (oxygen vacancy, impurities, lattice dislocations, etc.) can have a strong effect in the nucleation of the growth and the relaxation of the strain. In Figure 3(a,b), we show the comparison of AFM surface scan before and after the deposition of single SrO layer at 725°C. While the substrate has atomically flat TiO${\;}_{2}$ plane of about 200 nm width, the deposited layer shows a randomly distributed granular surface topology. This could originate by the fact that the surface diffusion lengths of the Sr and the O atoms are relatively small with respect to the width of the substrate terraces.

In order to overcome this small diffusion condition, we performed post-annealing of films at about 980°C for 5 h in oxygen. The result is a smoother surface as shown in Figure 3(c). After this annealing the surface roughness reduces from 0.12(1) to 0.06(1) nm, which may be a better condition for experiments in which the SrO is required as surface termination. This reorganization in more compact islands of the deposited layer suggests that with a partial coverage (20–80%) of the surface large self-organized patterns composed by SrO and TiO${\;}_{2}$-terminated islands could be obtained.

**Figure 4.**
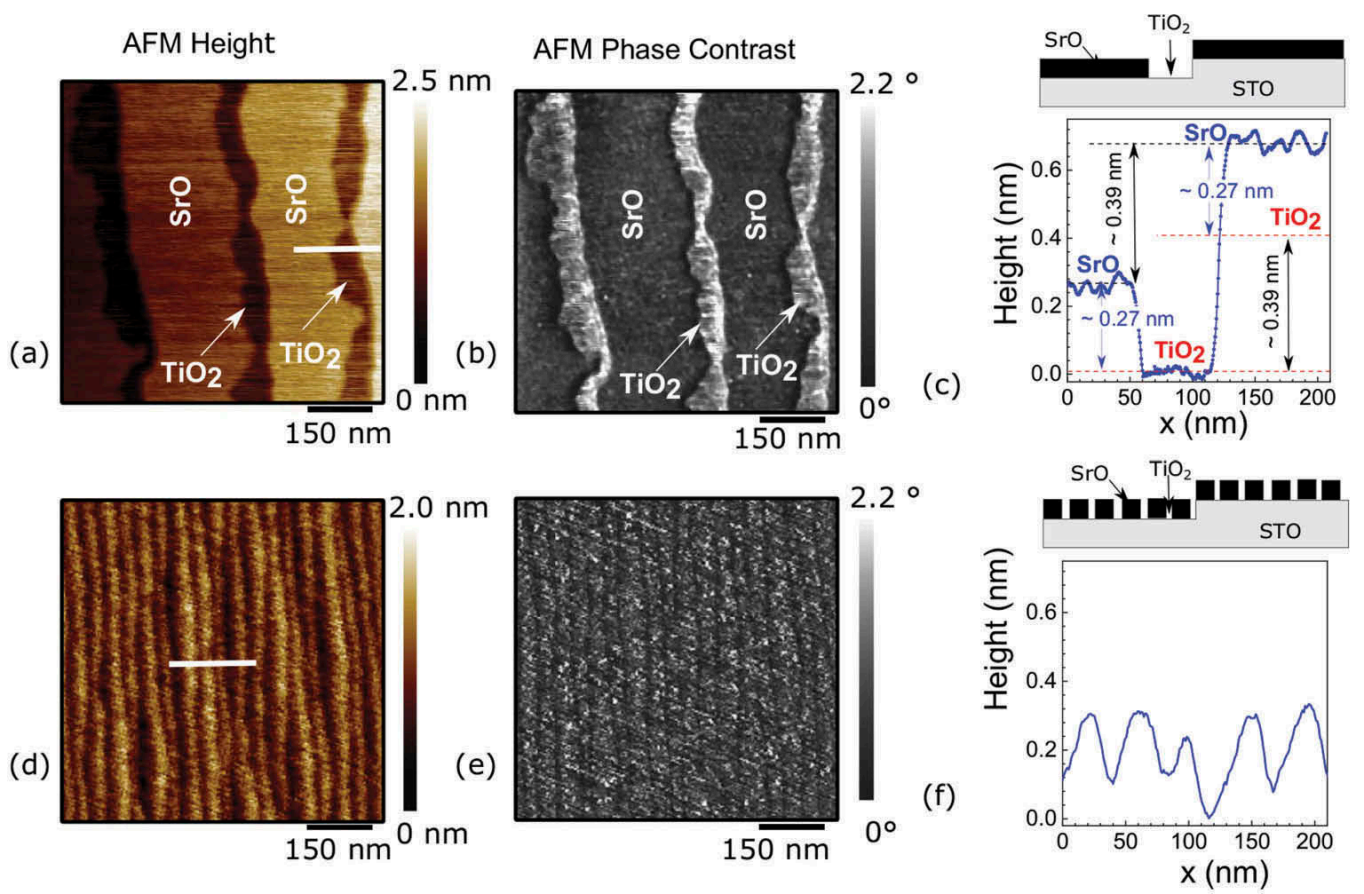
AFM characterization of two different cases of substrate surface partially covered by SrO (∼80%). (a) and (d) show AFM surface morphology of the two films, (b) and (e) show AFM phase contrast, and (c) and (f) show AFM profile in the region marked by the lines. Schematics of the surface cross-section are shown on top of the line profiles.

In order to test this idea, we deposited an amount of SrO which covers only about 80% of substrate. Here, we observed two different cases of self-organized structures. In the first case, as shown in Figure 4(a), relatively smooth and compact surfaces nucleate at each edge of the substrate terraces. Since the surface is covered partially, an uncoated TiO${\;}_{2}$ area is clearly visible in the AFM z-scan. This is also confirmed by the AFM phase-contrast imaging shown in Figure 4(b), where the SrO islands correspond to the dark gray and the TiO${\;}_{2}$ are represented by the white contrast.

The width of the TiO${\;}_{2}$ islands is about 40 nm near each of the pristine substrate steps. This pattern has been used by other authors for the selective growth of SrRuO${\;}_{3}$ stripes [8–10] with a typical width of 100–200 nm. Our experiment suggests that by increasing the SrO coverage one could potentially reduce the TiO${\;}_{2}$ terraces to a few nanometers, a condition which may be used for selective growth of quasi-one-dimensional nano-filaments. These uncoated regions allow us also to quantify the distance between the TiO${\;}_{2}$ and the SrO planes, and by performing a line profile analysis across 10 different steps we found an average value of 0.27(3) nm (see Figure 4(c)).^1^ This value is about 40% bigger than in the bulk of STO, and it is comparable to the height of 0.23 nm reported by Takahashi et al. [23]. SrO deposited on the surface has a different morphology respect to the ideal chemical termination that one could expect, and this could explain why the LAO/STO interface does not develop the 2DEG when the STO is terminated by SrO. It has been reported the possibility to grow STO by depositing alternating SrO and TiO${\;}_{2}$ planes [33–36]. In this special case, the TiO${\;}_{2}$ deposited on top of SrO forces the entire structure to reorganize like in the STO bulk, probably due to the strong covalent bonding of the oxygen octahedron which determine the lattice size. However here we found that a single SrO top layer behave different compared to the bulk and could be full of strain and defects.

The presence of strain is more clear in another case of self-organized pattern that we observed (see Figure 4(d)), where parallel stripes of about 30 nm width develop all over the surface. Since the lateral resolution of our AFM tip is about 5 nm, we could not resolve any empty space between the stripes in Figure 4(d), which is expected to be circa 80% of surface coverage, like the previous case in Figure 4(a). The relatively small stripe dimensions produce a continuous change of z during the scan. Since AFM scan in tapping mode become less precise in condition of constant deviation from a steady state, it is difficult to reveal properly the plateau of the underlay steps from the image. In fact we had to perform a second-order levelling [37] of this AFM image in order to recognize the stripes. This method produces flattening effects in the image. A schematic representation of the expected AFM profile for this case is present on top of Figure 4(f). Although similar initial substrate conditions were used, we observed two different SrO morphologies. We do not have a clear understanding of the mechanism that influences the two occurrences. While the miscut angle determines the width of the substrate terraces, another parameter may be relevant in order to understand the mechanism of formation of these self-organized patterns. We tentatively suggest that the direction of the substrate step edges (determined by the real cut) may be relevant. However, this direction cannot be easily controlled and it is random for different samples. In order to characterize the strain with substrate, we deposited a thicker film of about 8 nm of SrO. In this case, the behavior of the rock salt phase could be different with respect to the case of one single SrO plane; however, this film allows us to measure the strain generated at the interface by means of XRD.

**Figure 5.**
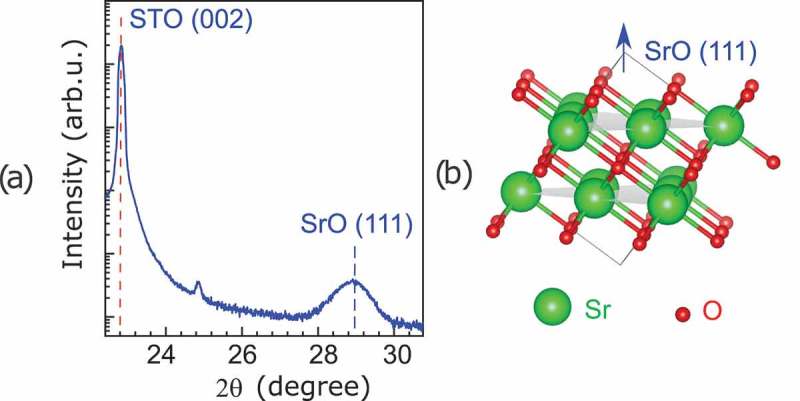
(a) XRD θ-2θ scan of SrO film with a thickness of about 8 nm deposited onto TiO${\;}_{2}$-terminated substrate. The peak present at 28.7° corresponds to the (111) reflection line of SrO. (b) Schematic representation of SrO along its [111] direction.

The XRD θ-2θ scan reported in Figure 5(a) shows only one SrO line at 28.7°.^2^ The closest reflection line of rock salt [29,30] is the (111); therefore, this is the preferential direction of the growth of SrO in our (100)-oriented substrate. From this line we could estimate (Bragg’s equation) that the distance between two adjacent Sr planes (Figure 5(b)) along the [111] direction is about 0.3096 nm, which indicates an elongation of about 4% with respect to the unstrained case (0.298 nm). By using the Scherrer equation and the full width at half maximum of this reflection line we estimated the film thickness a 8–9 nm. This value was confirmed by TEM analyses, shown later in this paper.

**Figure 6.**
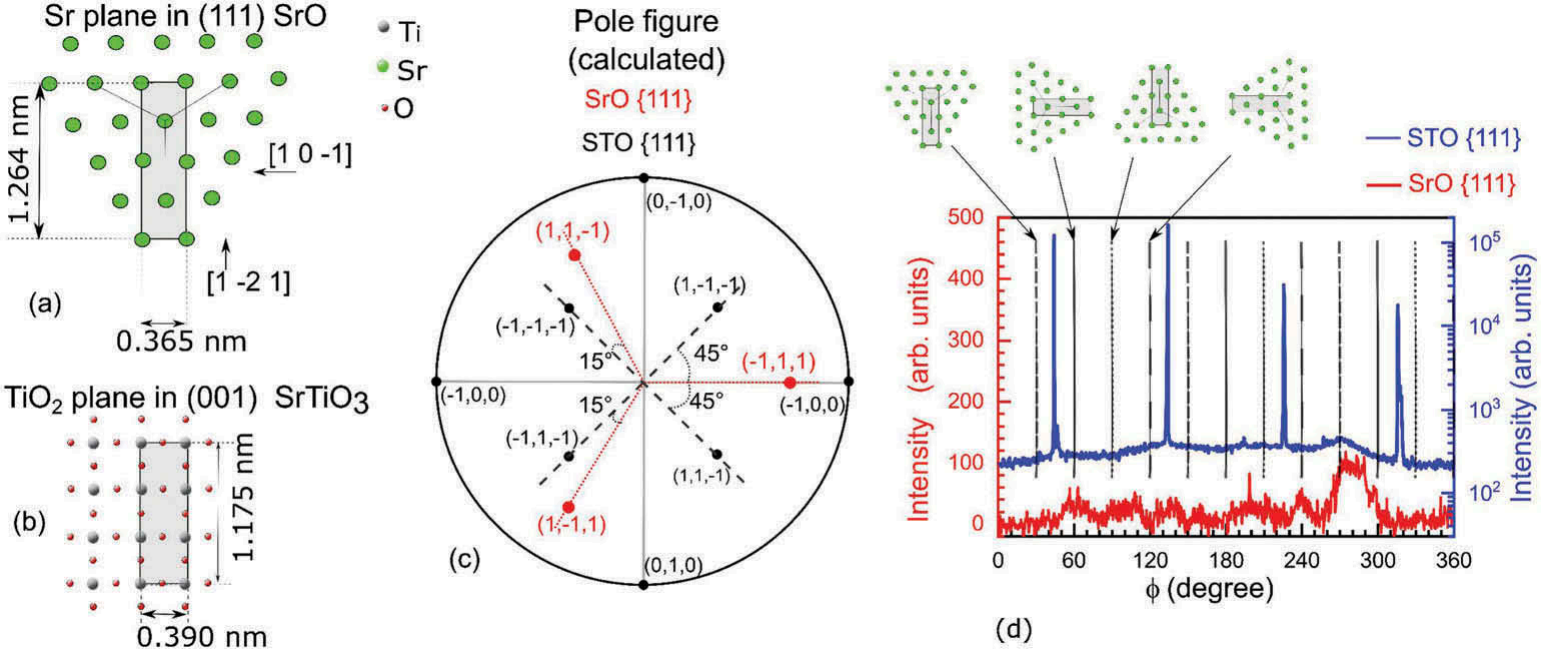
(a) Schematic representation of the Sr plane orthogonal to the [111] direction, and (b) TiO${\;}_{2}$ plane in (001)-oriented STO. (c) Theoretical pole figure. (d) Experimental ϕ-scan acquired around the STO{111} reflection (in blue at a tilt angle of 54.3°) and around the SrO{111} reflection (in red at a tilt angle of 70.53°).

In Figure 6, we attempt to describe how the SrO rock salt structure could adapt to the STO geometry, despite the large mismatch. The in-plane atomic distances are 0.390 and 0.365 nm for the STO and SrO, respectively. The [111] growth direction of SrO results in a honeycomb-like hexagonal in-plane structure (Figure 6(a)). Here, rectangular periodic structures of 1.264×0.365 nm${\;}^{2}$ could adapt (under strain) to the 1.175×0.390 nm${\;}^{2}$ of the TiO${\;}_{2}$ surface (which corresponds to 3 STO unit cells, see Figure 6(b)). The calculated pole figure of both STO and SrO undistorted lattices is shown in Figure 6(c). However, due to the fact that in-plane configuration of the substrate has a fourfold symmetry, every 90${\;}^{\circ }$ rotations of the SrO with respect to STO will produces a new possible match. Therefore, 3 peaks are expected for each of the four possible matches resulting in 12 equally spaced peaks for the SrO. The ϕ-scans of the SrO{111} and STO{111} planes are reported in Figure 6(d).

The SrO ϕ-scan, results low in intensity, and due to a poor signal-to-noise ratio we could see only some of the 12 expected lines. The model presented above is just a possible scenario for the in-plane alignment. However we cannot exclude the presence of interface disorder due to defects formation at the interface [31,32]. The large lattice mismatch with the STO underneath may cause dislocation formation and compressive in-plane strain which could explain the 4% out-of-plane elongation of SrO along the [111] direction measured by XRD.

**Figure 7.**
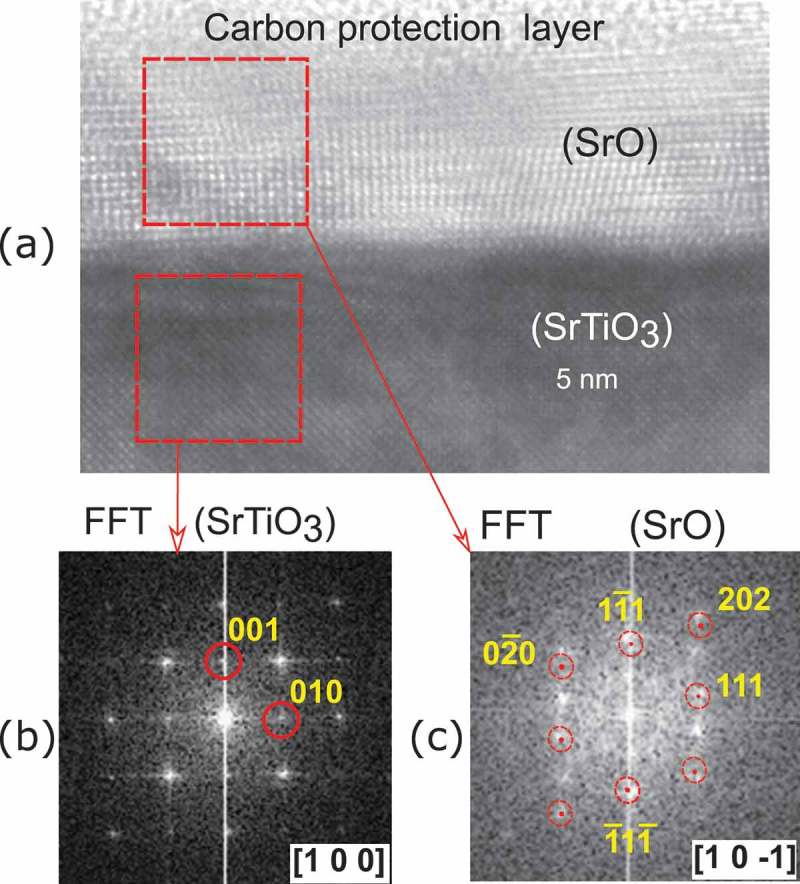
(a) TEM image of SrO film grown on STO. Fast Fourier transform of TEM patterns of SrTiO${\;}_{3}$ (b) and SrO (c) selected regions. Here (111)-oriented SrO domain is clearly visible with the following orientation relations: out-of-plane [0 0 1${]}_{STO}∥[$1 1 1${]}_{SrO}$ and in-plane [ 1 0 0 ${]}_{STO}∥[$1 0 −1${]}_{SrO}$.

TEM analysis shows that our thicker film is composed by *c*-axis oriented nano-domains separated by extended defect regions like stacking faults or more general dislocations (Figure S1 in supplemental data). The in-plane width of the domains is about 20–30 nm, similar in size to the SrO stripes shown in Figure 4(d). Therefore the dimensions of the self-organized pattern observed in the initial layer could be related to a strain with substrate which remains during the growth of thicker films. A representative selected region is shown in the TEM micrograph of the Figure 7(a). The lamella was imaged along the [100] direction of STO substrate as shown by the fast Fourier transform (FFT) on Figure 7(b).

From the FFT analysis of the film shown in Figure 7(b,c), we estimated a distance of two adjacent Sr planes of about 0.31(2) nm, and we found the following orientation relations between substrate and film: out-of-plane [ 0 0 1${]}_{STO}∥[$1 1 1${]}_{SrO}$, and in-plane [ 1 0 0 ${]}_{STO}∥[$ 1 0 −1 ${]}_{SrO}$ (see Figures S2 and S3 of supplemental data for more details). These orientations are in agreement with the model presented in Figure 6(a,b) and are consistent with the XRD pattern of Figure 5(a). Hence, we deduce that the majority of our film consist of these strained nano-domains, and because of interface disorder induced by the lattice mismatch we cannot exclude the presence of other domain orientations in a volume fraction difficult to estimate.

## 4. Conclusions

We investigated the growth of SrO onto TiO${\;}_{2}$-terminated STO. Although similar initial substrate conditions were used, we observed two cases of special self-organized patterns that form when we deposit less than one layer (coverage of about 80%). In one case, a relatively smooth and compact surface nucleates at one edge of TiO${\;}_{2}$ terraces and leaves empty space before the next terrace step. This particular topology allowed us to measure that the average height of deposited SrO is about 0.27(3) nm. A similar value has been observed already by Takahashi et al. [23] and it is substantially different from 0.19 nm which is the distance between SrO-TiO${\;}_{2}$ planes, expected in STO bulk.

In the other case, we observed self-organized parallel stripes islands of about 20–30 nm width. Here, we assume that the formation of this stripe-morphology is related to a large strain with the substrate which can be released by forming nano-sized domains of about 20–30 nm. In fact, XRD and TEM showed that SrO grows preferentially along its [111] direction. In such orientation, the SrO may be described as alternating planes of oxygens and strontium atoms as shown in Figure 5(b), and its lattice size matches better with the substrate. This geometry may explain the SrO surface granularity measured by AFM.

We demonstrate that by controlling the SrO/TiO${\;}_{2}$ ratio, one can obtain self-organized patterns of the two terminations. This could be potentially used for selective growth of quasi-one-dimensional nano-filaments of other materials.

Even if lattice mismatch at the interface with STO is reduced by the (111) growth, an out-of-plane elongation of 4% was clearly observed by TEM and XRD along this direction. We propose that this large strain is also responsible for self-organized patterns formed in the initial growth of SrO.

It has been reported the possibility to grow STO by depositing alternating SrO and TiO${\;}_{2}$ planes [33–36]. In this special case, the TiO${\;}_{2}$ deposited on top of SrO forces the entire structure to organize like in the STO bulk, probably due to the strong covalent bonding of the oxygen octahedron which determine the lattice size. However, we found that a single SrO top layer is full of strain, intrinsically different from the bulk case, and cannot be considered an atomically flat chemical termination.

## Notes

1. An instrumental AFM offset across the two different materials was estimated to be about 0.01 nm by measuring a selected TiO${\;}_{2}$-SrO-TiO${\;}_{2}$ intermediate step in special substrates, similar to the cases reported in literature [8,22].

2. The XRD scan reported shows the presence of a strontium hydroxide phase (although small in intensity) only observed for the thicker film. No evidence of this phase was found for thinner films of SrO. Moreover, the XRD measurements were repeated after about 5 months in order to check the stability of the SrO film in air. No visible changes in the intensity and peak shape were detected.
