# Supplementary material for: Structural properties of ultrathin SrO film deposited on SrTiO3
Source: Sci Technol Adv Mater. 2019 May 20;20(1):456–63. doi: 10.1080/14686996.2019.1599693 (PMC6542177; doi:10.1080/14686996.2019.1599693)
Supplement: Supplemental Material [file TSTA_A_1599693_SM1925.pdf]

1 Supplemental material

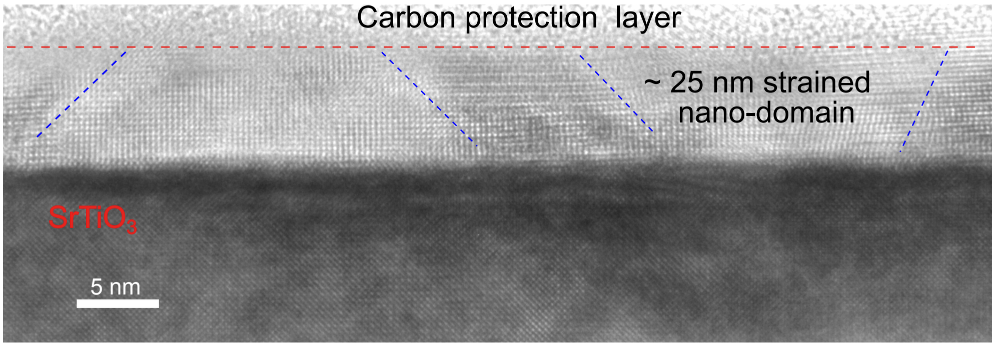

S1: TEM image of SrO film grown on STO. Strained nano-domains of about 25 nm width are confined between dislocations.

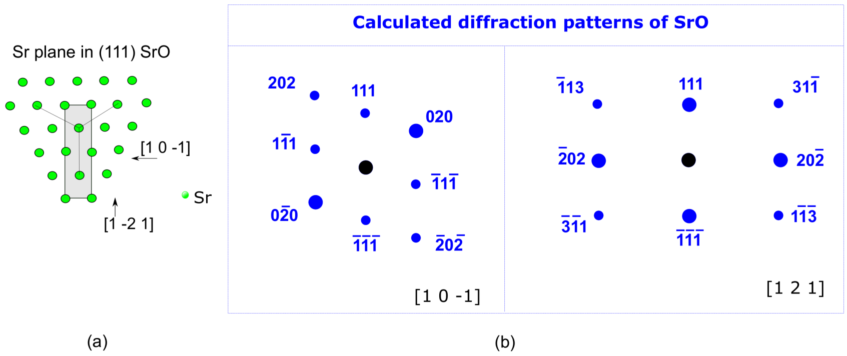

S2: (a) Schematic representation of the Sr plane orthogonal to the [111] direction. (b) TEM diffraction patterns of SrO calculated for the [1 0 -1] and [1 -2 1] directions.

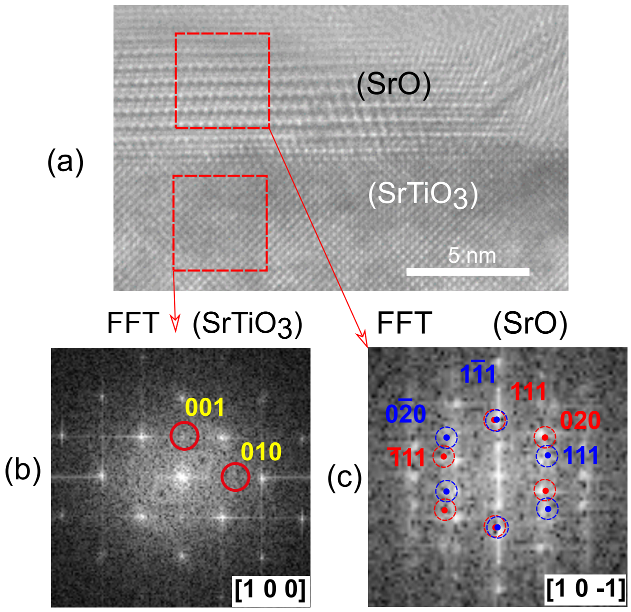

S3: (a) TEM image of SrO film grown on STO. Fast Fourier transform of TEM patterns of selected regions of $SrTiO_3$ (b) and SrO (c). Due to the lamella thickness ($\sim 100\,$nm) we observe the superimposed diffraction patterns of two SrO domains imaged along [1 0 -1] direction.
